# The Availability and Safety Study of Remimazolam Besylate for Injection on Sedation of ERAS Patients Under Mechanical Ventilation in ICU: Protocol for a Randomized, Open-Label, Controlled Trial

**DOI:** 10.3389/fmed.2021.735473

**Published:** 2021-11-05

**Authors:** Shengjun Liu, Longxiang Su, Bo Zhang, Huaiwu He, Zunzhu Li, Qi Li, Qianlin Wang, Fang Smith, Yun Long

**Affiliations:** ^1^Department of Critical Care Medicine, State Key Laboratory of Complex Severe and Rare Diseases, Peking Union Medical College Hospital, Peking Union Medical College, Chinese Academy of Medical Sciences, Beijing, China; ^2^Department of Pharmacy, Peking Union Medical College Hospital, Chinese Academy of Medical Sciences, Beijing, China; ^3^Birmingham Acute Care Research Centre, Institute of Inflammation and Aging, University of Birmingham, Birmingham, United Kingdom

**Keywords:** Remimazolam Besylate, ERAS, mechanical ventilation, intensive care unit, sedation

## Abstract

**Introduction:** The most common physiological and psychological disorders associated with critical care patients are pain and anxiety. Sedatives and analgesics are commonly used to relieve these symptoms. However, the adverse effects of sedatives and analgesics are common and inevitable. As a new type of sedative drug, limited number of trials are available to evaluate Remimazolam Besylate's availability and safety compared with propofol.

**Methods:** This study is a single center, randomized, open-label, controlled trial. A total of 84 patients who meet ERAS criteria and receive mechanical ventilation in ICU, aged ≥18 years old will be included. Patients will be randomized (1:1) into two groups: Remimazolam Besylate group and Propofol group. The Primary outcomes includes satisfaction rate of sedation and incidence rate of major clinical events. Secondary outcomes including incidence of delirium, time to weaning and extubation, Difficulty of nursing RASS, BIS and PI, 28-days survival, side-effect and vital signs during medications, total dose and dose per kilogram body weight of analgesic and sedatives and incidence of rescue therapy in experimental group.

**Ethics and Dissemination:** This trial has been approved by the ethics boards of Peking Union Medical College Hospital. Recruitment began in January 2022 and will continue until June 2022. Dissemination plans include presentations at scientific conferences, scientific publications, stakeholder engagement efforts and presentation to the public via lay media outlets.

**Clinical Trial Registration:** www.ClinicalTrials.gov, identifier: NCT04947345

## Introduction

### Background

Pain and anxiety are the most common physiological and psychological disorders associated with critical care patients. Organ dysfunction caused by severe diseases in ICU patients, combined with environment and various invasive treatments can produce strong pain and psychological stress, which may directly affect the respiratory, circulatory and immune functions of patients (1). Analgesia, sedation and humanistic care can reduce stress response, relieve tension, and reduce the incidence of delirium (2, 3). However, irregular analgesia and sedation treatments are not only ineffective, but may also cause adverse reactions.

Currently commonly used sedative drugs in clinical practice include midazolam, propofol, and dexmedetomidine. Midazolam has a good sedative effect and has little effect on the respiratory and circulation. At the same time, it can be antagonized by flumazenil. However, midazolam has a slower onset of action and a longer awakening time. As the infusion duration increases, it is easy to cause accumulation. Although propofol has a good sedative effect and can wake up quickly, it is prone to respiratory/circulation depression, and no antagonist was found suggest potential risks. Dexmedetomidine has a good sedative effect and has a small effect on respiratory, however, the pharmacological mechanism of dexmedetomidine determine its slow onset and negative effect on heart rate and blood pressure.

As a new type of sedative drug, Remimazolam can be rapidly metabolized by tissue esterase in the body to produce inactive metabolites, so that its sedative effect will quickly fade. In a phase I single-center randomized double-blind study, researchers found that Remimazolam can reach the peak blood concentration 1 min after intravenous infusion, the metabolism is rapid, and continuous infusion has almost no accumulation (4). Studies also show that Remimazolam has little effect on respiratory/circulation system. Blood pressure and heart rate fluctuate little during the injection of Remimazolam (5). The pharmacokinetics research on healthy volunteers shows its clearance is not related to weight, which provide more sedative option for high weight patients in ICU.

The PK/PD study of Remimazolam in healthy people has been carried out lately (6, 7). As for clinical applications, the effectiveness and safety evaluation of Remimazolam was carried out in phase IIb clinical study of colonoscopy sedation in 2015 (8), bronchoscopy sedation in 2019 (9), anesthesia induction and maintenance in 2020 (10). These studies showed Remimazolam has certain advantages in the effectiveness and safety of sedation.

### Objectives

This study is designed to verify the hypothesis that compared with propofol, Remimazolam Besylate for Injection has non-inferiority availability and safety effect on sedation of ERAS patients under mechanical ventilation in ICU. The primary outcome is satisfaction rate of sedation and incidence rate of major clinical events. For availability evaluation, the secondary outcome includes incidence of delirium, time to weaning and extubation, difficulty of nursing, RASS, BIS and PI during medications. For safety evaluation, the secondary outcome includes 28-days survival, side-effect and vital signs during medications, total dose and dose per kilogram body weight of analgesic and sedatives and incidence of rescue therapy in experimental group.

## Methods and Analysis

### Trial Design/Study Setting

A prospective, single-center, randomized controlled trial in patients who meet the inclusion criteria. This study is performed in Intensive Care Unit (ICU) in Peking Union Medical College Hospital. All staffs that participate in this study have professional licenses and certificated in Peking Union Medical College Hospital. The approval number of ethics committee of Peking Union Medical College Hospital is 20-JS-2814.

### Eligibility Criteria

#### Inclusion Criteria

- Meet criteria of enhanced recovery after surgery (ERAS);
- Mechanical ventilated when enrolled and have estimation of more than 10 h of mechanical ventilation in ICU;
- Aged between 18–75 years old and 18 ≤ BMI ≤ 30 kg/m^2^;
- Clearly know the purpose and objective of this clinical study and voluntarily enrolled.

Criteria of ERAS: (1) without dysfunction of nervous system or Glasgow Coma Score >12; (2) a satisfied glucose level (random blood glucose <11.1 mmol/L during screening stage) for diabetes mellitus patients; (3) Without acute coronary syndrome in recent 6 months; (4) Without bradycardia and third-degree atrioventricular block (except for patients with pacemaker) during screening stage; (5) systolic blood pressure >90 mmHg with no usage of vasoactive agent during screening stage; (6) without mental illness (schizophrenia, depressive disorder), cognitive dysfunction (identified by MMSE), epilepsy, history of abuse of psychotropic, or anesthesia medication; (7) without disorder of coagulation function (PT/INR/APTT > 1.5 × upper limit), bleeding tendency (active peptic ulcer), under treatment of thrombolysis and anticoagulant; (8) without disorder of liver function (ALT/AST > 2 × upper limit and total bilirubin > 1.5 × upper limit); (9) without disorder of renal function (Creatine or BUN/Urea > 1.5 × upper limit); without dialysis patients.

#### Exclusion Criteria

- Allergy to component of Remimazolam besylate for injection;
- Woman in gestation and lactation period;
- Enrolled in other clinical trials in recent 3 months;
- Patients who needs deep sedation levels (RASS < −2) based on clinical considerations;
- Other circumstance that identified by researchers that do not suitable for this clinical trial.

## Interventions

Patients will be randomized as soon as they arrive at intensive care unit, the end point of this study is 28 days after patients depart from intensive care unit.

### Experimental Group: Remimazolam Besylate

Patients should be given study drugs after randomization. Syringe pump is used to administer medication. Loading dose of Remimazolam is 0.02–0.1 mg/kg, which is administered for <1 min initially. Maintaining dose is 0.2–1 mg/kg/h. Sedative target is RASS between 0 and −2 points. Excepts for fentanyl and Remimazolam, no analgesic or sedative could be used during the experimental time period.

### Positive Control Group: Propofol

Patients should be given study drugs after randomization. Syringe pump is used to administer medication. Loading dose of propofol is 0.2 mg/kg, which is administered for <1 min initially. Maintaining dose is 0.3–4.0 mg/kg/h. Sedative target is RASS between 0 and −2 points. Excepts for fentanyl and propofol, no analgesic or sedative could be used during the experimental time period.

### Rescue Therapy for Experimental Group

During the treatment of the experimental group, if the RASS cannot be maintained at 0 to −2 points at the maximum Remimazolam maintenance dose of 1 mg/kg/h for more than 3 min, a loading dose of propofol (0.2 mg/kg) can be given intravenously. If RASS fails to satisfy after three loading doses of propofol, Remimazolam is discarded and 0.3–4.0 mg/kg/h of propofol are used as rescue therapy for experimental group.

## Outcomes

### Primary Outcomes

Validity endpoint is satisfaction rate of sedation, which equals to proportion of time in target sedation range (RASS score, 0 to −3) without use of rescue therapy of the total duration of study drug infusion. Safety endpoint is the incidence of hypotension (SBP < 90 mmHg), the incidence of hypoxia (SpO_2_ < 90%), and the incidence of bradycardia (HR < 75% baseline HR or HR < 50 bpm).

### Secondary Outcomes

For validity evaluation, the secondary outcome includes incidence of delirium (subjects with delirium/total number of subjects × 100%) by 12 and 24 h after entering ICU. Time to weaning and extubation are recorded. Difficulty of nursing is evaluated by TISS-28 scoring system. RASS, BIS and PI are recorded every 30 min during medications. For safety evaluation, the secondary outcome includes 28-days survival, vital signs during medications, total dose and dose per kilogram body weight of analgesic and sedatives and incidence of rescue therapy in experimental group, side-effect report from usage of medications to 28 days after patients depart from intensive care unit. Side-effect is defined as hypotension, bradycardia, elevation of bilirubin, abnormal urine (Elevated urinary leucocyte/ketone body/bilirubin) in this study.

## Sample Size Planning

In this study, propofol was used as a positive control. The hypothesis is compared with propofol, Remimazolam Besylate for Injection has non-inferiority satisfied rate of sedation on ERAS patients under mechanical ventilation in ICU. Setting inspection level: α = 0.05, 1–β = 80%, the non-inferiority threshold is 10%. Subjects in experimental group and control group is 1:1. According to the results of previous clinical trials, the target sedation rate of the propofol group is 82% (11), assuming that the target sedation rate of the Remimazolam Besylate group is 92%, a total of 70 subjects (35 for experimental group and 35 for control group each) should be enrolled to meet inspection level. Sample size calculations allow for 15% loss of cases, a total of 84 subjects (42 for experimental group and 42 for control group each) are enrolled in this clinical trial.

## Recruitment

All participants will be recruited in hospital settings between the time of admitted and the time of surgery. Patients who both meet criteria of enhanced recovery after surgery (ERAS) and may potentially admitted to ICU are considered as potentially eligible patients. A member of the research team will approach the patient and/or their legally authorized representative (based on the law of the People's Republic of China) before the surgery. A brief screening evaluation will be conducted. If the patients fit all eligibility criteria, the purpose, gain and risks of this clinical trial will be detailed delivered to the patients and/or their legally authorized representative. A written informed consent is required to be finished if patient AND their legally authorized representative agree to join in this clinical trial. For patients who are too sick or who are not competent to give their own permission to enter the study, consent will be obtained from the patient's legally authorized representative (based on the law of the People's Republic of China).

## Allocation

Randomization will be carried out on the day of surgery immediately after the patients entered ICU and will be performed centrally through an online electronic data management system after confirmation with researchers that the patient is suitable for randomization. Participants will be randomly assigned to one of the two groups in a 1:1 ratio. Participants and their clients, researchers who are responsible for assessment, data recording and statistical analysis will be blinded to treatment assignment. The researchers will not be blinded to treatment assignment.

## Data Management

Data will be collected using an online data collection form via a secure, password-protected platform with predefined data fields. Based on the PUMC Intensive Care Medical Information System and Database (PICMISD), all bedside instruments, including monitors, mechanical ventilators, and blood gas analyzers are connected into a central server to achieve real-time data acquisition and synchronization. The clinical data of the patients involved in this study included (1) monitors parameters: respiratory rate (RR), heart rate (HR), mean arterial pressure (MAP), body temperature, central venous pressure (CVP), perfusion index (PI), bispectral index (BIS). (2) Data of arterial blood gas analysis (ABG), including lactate level, pO2, GAP, ScvO_2_. (3) Data of Biochemical laboratory analysis and evaluation scale. (4) Medication: usage and drip speed of vasoactive agent, liquid usage and balance, usage and drip speed of sedatives and analgesics. All data used in the statistical analyses were collected during the clinical trial. All identifiable data collected, processed and stored for the purposes of the project will be kept confidential at all times and comply with Good Clinical Practice guidelines for Research and the General Data Protection Regulation. The research project will be monitored closely by a certified external auditor to ensure that study activities are carried out in accordance with the protocol, good clinical practice and applicable regulatory requirements. Data quality will also be audited.

## Pharmacokinetic Sampling

A total of <20 mL of whole blood samples are collected in heparin tubes during the clinical trial. Opportunity blood sampling is used as sampling strategy, which is, 5, 15, and 30 min after initial dosing and 0, 10, and 30 min after changing pump speed. After direct centrifuging, a plasma aliquot is stored at −80°C until analysis. Remimazolam Besylate concentrations are measured using HPLC-MS/MS analysis.

## Statistical Methods

### General Statistics

A descriptive analysis of the data will be carried out. Qualitative variables will be represented by a frequency distribution of the percentages for each category, and quantitative variables will be explored using the Kolmogorov-Smirnov conformity test. The association between factors will be investigated using hypothesis contrast tests, with a comparison of proportions when both variables are qualitative (χ^2^, Fisher's exact test), a comparison of mean when one of them is quantitative (Student's *t*-test, analysis of variance (ANOVA), and the Mann-Whitney *U*-test or the Kruskall-Wallis test if they do not follow a normal distribution) and a bivariate correlation (Pearson correlation coefficient) when both are quantitative or the Spearman correlation if the conditions for application of the former are not met. For comparisons in related samples when one of them is quantitative, Student's *t*-test and/or ANOVA will be used (Wilcoxon or Friedman's test if they do not follow a normal distribution). The analysis will be completed using multivariate regression models. A survival analysis will be performed using the Kaplan-Meier method, and the log-rank test will be used for survival comparisons between groups. Effects will be considered to be significant with a *p* value of < 0.05.

### Pharmacokinetics Models

Non-linear mixed effects modeling (NONMEM) was used to analysis pharmacokinetic feature of Remimazolam Besylate for Injection in patients in ICU. Based on simulation of pharmacokinetic pattern, a recommended dosage and medication strategy for ERAS patients under mechanical ventilation in ICU is expected to carry out.

## Patients and Data Safety Monitoring

Besides routine critical care, a full-time certificated doctor and certificated nurse are responsible for observe patients and draw blood sample. A medical advisor team within the researcher team is 24-h online for any consulting related to the medication. All serious adverse events, as well as all non-serious adverse events that are unexpected and judged to be related to the study treatment, will be recorded in the study database and reported as required to Ethics Committee of Peking Union Medical College Hospital.

## Harms

The risks and harms associated with this study are low. The risk of a breach of confidentiality is small and all possible efforts have been taken to ensure the security of study data and minimize the risks of accidental disclosure of identifiable data elements. The medical risks for participation in this study do not go beyond those risks typically associated with sedation treatment in in routine clinical care. Beyond the study consent, patients will also undergo standard procedural consent to discuss the risks and benefits of this clinical trial. If major clinical events or side-effect occurs, researchers will report to ethics committee and medical department of Peking Union Medical College Hospital. A quick response team will be formed before the clinical trial begin. Once major clinical events or side-effect occur, the quick response team will handle the clinical treatment, including emergency rescue and injuries assessment. Besides, researchers will purchase clinical trial insurance for each subject, which may give compensation for any harm happened in this clinical trial.

## Ethics Approval and Informed Consent

Ethical approval on this protocol (v1.2) was obtained on 23rd March 2021 by the Ethics Committee and Medical department of Peking Union Medical College Hospital (20-JS-2814). Any substantial amendment made to the protocol by the coordinating investigator is sent to the Ethics Committee of Peking Union Medical College Hospital for approval, prior to implementation. All participants will give written informed consent prior to data collection and medication.

## Dissemination

Study results will be submitted for publication in peer-reviewed medical journals and presented to patients, healthcare professionals, and the public during (inter)national meetings. The full study protocol and the informed consent form are available from the corresponding author. After study completion, the participant-level dataset and statistical code will be available on reasonable request.

## Discussion

To our knowledge, this is the first clinical trial that investigate availability and safety of Remimazolam Besylate as sedatives in ERAS patients under mechanical ventilation in ICU, especially for process of weaning and extubation of mechanical ventilated patients. Compared with midazolam, Remimazolam Besylate has a short half-life period (4). It is an ester-based drug that rapidly hydrolyzed in the body by tissue esterase to an inactive carboxylic acid metabolite (12), which may relieve the burden of liver and kidney. Hence, unlike propofol and midazolam, physicians don't have to consider accumulation effect of sedatives.

## Ethics Statement

The studies involving human participants were reviewed and approved by Ethics committee of Peking Union Medical College Hospital. The patients/participants provided their written informed consent to participate in this study. Written informed consent was obtained from the individual(s) for the publication of any potentially identifiable images or data included in this article.

## Author Contributions

YL took responsibility for the integrity of the work as a whole. SL, HH, and BZ were responsible for study design and conception, drafted the manuscript, and carried out the clinical trial. ZL, QL, and QW were responsible for carrying out the clinical trial. LS was responsible for calculating sample size and drafted the manuscript. All authors revised the manuscript for important intellectual content.

## Funding

This work was funded by China Health Information and Health Care Big Data Association Severe Infection Analgesia and Sedation Big Data Special Fund (Grant No. Z-2019-1-001), China International Medical Foundation Analgesia and Sedation Special Fund (Grant No. Z-2017-24-2028-01), and Excellence Program of Key Clinical Specialty of Beijing in 2020. Beijing Municipal Science and Technology Commission (Grant No. Z201100005520051).

## Conflict of Interest

SL, LS, BZ, ZL, QL, QW, HH, and YL are employed by Peking Union Medical College Hospital. FS is employed by Institute of Inflammation and Aging Professor in Anesthesia, Critical Care and Pain, University of Birmingham. The authors declare that this study received funding from (1) China Health Information and Health Care Big Data Association Severe Infection Analgesia and Sedation Big Data Special Fund (2) China International Medical Foundation Analgesia and Sedation Special Fund. (3) Excellence Program of Key Clinical Specialty of Beijing. The funders had the following involvement in the study: Pay for the publishing charges and paper polish charges. This study also received funding from Yichang Humanwell Pharmaceutical Co. Ltd. The funder had the following involvement in the study: Provide medication needed for the study. All funders was not involved in the study design, collection, analysis, interpretation of data, the writing of this article or the decision to submit it for publication.

## Publisher's Note

All claims expressed in this article are solely those of the authors and do not necessarily represent those of their affiliated organizations, or those of the publisher, the editors and the reviewers. Any product that may be evaluated in this article, or claim that may be made by its manufacturer, is not guaranteed or endorsed by the publisher.
